# Response to stress in biological disorders: Implications of stress granule assembly and function

**DOI:** 10.1111/cpr.13086

**Published:** 2021-06-25

**Authors:** Lingjuan Wang, Weina Yang, Bin Li, Shuiqiao Yuan, Fengli Wang

**Affiliations:** ^1^ Institute Reproductive Health Tongji Medical College Huazhong University of Science and Technology Hubei China; ^2^ Tianjin Medical University General Hospital Tianjin China; ^3^ State Key Laboratory of Environmental Chemistry and Ecotoxicology Research Center for Eco‐Environmental Sciences Chinese Academy of Sciences Beijing China

**Keywords:** assembly, biological disorders, germ cells, heat stress, stress granules

## Abstract

It is indispensable for cells to adapt and respond to environmental stresses, in order for organisms to survive. Stress granules (SGs) are condensed membrane‐less organelles dynamically formed in the cytoplasm of eukaryotes cells to cope with diverse intracellular or extracellular stress factors, with features of liquid‐liquid phase separation. They are composed of multiple constituents, including translationally stalled mRNAs, translation initiation factors, RNA‐binding proteins and also non‐RNA‐binding proteins. SG formation is triggered by stress stimuli, viral infection and signal transduction, while aberrant assembly of SGs may contribute to tissue degenerative diseases. Recently, a growing body of evidence has emerged on SG response mechanisms for cells facing high temperatures, oxidative stress and osmotic stress. In this review, we aim to summarize factors affecting SGs assembly, present the impact of SGs on germ cell development and other biological processes. We particularly emphasize the significance of recently reported RNA modifications in SG stress responses. In parallel, we also review all current perspectives on the roles of SGs in male germ cells, with a particular focus on the dynamics of SG assembly.

## Graphical abstract

Schematic illustration for heat stress in male germ cells. Heat stress induces stress granule (SG) formation by phosphorylating eIF2α, recruiting RNA binding protein (DAZL, BOULE, RACK, MAGE‐B2) and ultimately protecting normal spermatogenesis from germ cell apoptosis. eIF2α (eukaryotic initiation factor‐2alpha), MAPK (mitogen‐activated protein kinases), DAZL (deleted in azoospermia‐like), BOULE (a founding member of the DAZ gene family), RACK (receptor for activated protein kinase C), MAGE‐B2 (testis‐specific protein)

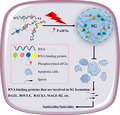

## 1 INTRODUCTION

When exposed to an adverse stimulus, regular biological processes would be perturbed, ultimately resulting in impaired fertility^1^, ^2^ and other disorders.^3^, ^4^ Protein translation is one of the most sophisticated biological processes in eukaryotic cells. In coping with stressful environments, eukaryotic cells reprogram translation mechanisms and specialize in the synthesis of functional proteins to adapt to the changing conditions for survival. The pivotal pathway in response to external stimuli is the formation of stress granules (SGs) comprising a large amount of untranslated mRNA to suspend mRNA translation.^5^

Stress granule is a highly conserved and predominant type of cytoplasmic ribonucleoprotein (RNP) granule, mainly composed of non‐translating mRNAs and proteins.^6^ The α‐subunit of eukaryotic initiation factor 2 (eIF2α) is phosphorylated by an upstream kinase (eg protein kinase R [PKR]) when cells are stimulated by environmental factors, thereby impeding the assembly of the 43S complex and subsequently delaying mRNA translation initiation.^7^, ^8^ At this point, the translation‐suspended mRNA and associated protein aggregates and forms SG. Upon removal of the stimulus, SGs depolymerize through microtubules and dynein to release the wrapped mRNA and proteins, and restore normal mRNA translation.^9^ Recent studies have demonstrated that dynamical SG is a mechanism involved in cellular protection.^10^, ^11^, ^12^ When disturbed by specific server adverse factors, SGs can promote cell apoptosis through stress‐activated pathways.^13^ Thus, SGs are considered as the essential structure for normal cellular biological events. However, it remains to be elucidated how SGs assemble and participate in spermatogenesis, which has also attracted wide attention regarding male fertility.

Germ cells have the crucial fundamental role in the multicellular organism.^1^ Compared with somatic cells, germ lines contain genetic information that can be passed on continuously generation after generation and thus must be protected from environmental forces to avoid drastic genetic inaccuracy.^14^ Given the distinct and unique properties, it is reasonable that germ cells have evolved specific cellular mechanisms to counter stresses. Stress responses function not only in cell survival but also in maintaining gamete quality that, once be damaged, would result in developmental arrest and even severe birth disorders.^14^ Germline is complicatedly regulated in gene expression, with abundant maternal mRNAs accumulating in oocytes, most of which are not translated until fertilizing.^15^ Reasonably, stress responses may be specific in oocytes, and some appear to be unique to germ cells.

To better understand how the male reproductive system respond to environmental influences and the role of SGs in male germ cells, we review advanced discoveries in this field and provide some perspectives on future research. More specifically, we summarize current views on the role of SG components in male germ cells and focus on the dynamic assembly of SGs, which is important for identifying other structures and the factors affecting reproduction, further expanding our understandings of human fertility. In addition, more in‐depth insights into regulated and protective mechanisms to defend against environmental forces in germ cells are discussed in this review, which will provide a reference for the clinical treatment of male infertility. Alternatively, reports on the formation of SG and its biological significance in recent years are also summarized, providing clues and research directions for future research in related fields, such as inflammatory response,^16^ degenerative disease ^17^ and cancer.^18^, ^19^

## 2 STRESS GRANULES FORMATION

### 2.1 Stress granules

Ribonucleoprotein granules (RNPs), non‐membrane‐coated organelles containing RNA and protein condensates in eukaryotic cells, are independent high‐order subcellular organelles composed of multiple biomolecules. They are ubiquitously presented in both the cytoplasm and nuclei, shown as puncta with a diameter of 0.1‐4 microns.^20^ RNP granules have been involved in many biological processes, including synaptic plasticity in neurons and maternal mRNA storage in oocytes.^21^ Cytoplasmic RNPs mainly include SGs, P‐bodies, germ cell granules, and neuronal granules, whereas nuclei RNP particles include paraspeckles, the nucleolus, Cajal bodies,^22^, ^23^ among which SGs have been widely investigated.

Stress granule is a brilliant way for cells to react to external stimuli. Certain adverse conditions (non‐biological stimuli such as heat shock, viral infection, oxidative stress, ultraviolet radiation and hypoxia) trigger SGs assembly in cells, which is a major adaptive defence mechanism of cell adaptation.^22^, ^24^ SGs are multimolecular polymers of the pre‐translational complex of stasis, preventing the accumulation of misfolded proteins.^10^ Huang et al^25^ demonstrated SGs formation can be induced by five different chemicals representing different stress conditions, including oxidative stress (sodium arsenite, hydrogen peroxide), osmotic stress (sorbitol, sodium chloride) and clotrimazole.

Stress granule is a type of the highly conserved cytoplasmic RNP granules, generally containing untranslated mRNA, ribosome subunits, the RNA‐binding proteins (eg Ras GTPase‐activating protein binding protein [G3BP1], T‐cell intracellular antigen‐1 [TIA‐1]) and various translation initiation factors, which consist of the stagnant 48S preinitiation complex.^20^ SG‐like RNPs containing a large amount of untranslated mRNA were found in neurons and embryos.^26^ SGs cannot form when mRNAs are captured by polysomes.^27^ These evidences indicate that ribosome‐related mRNAs cannot be recruited to SGs. Moreover, it has been observed that SG‐related proteins (TIA‐1/TIAR) and specific mRNAs (such as TOP mRNAs) participate in translational initiation steps, which further reveal that SGs are a collection of translationally arrested mRNPs.^28^ However, the compositions of SGs are variable under exposure in different adversities. Taking *Saccharomyces cerevisiae* as an example, eIF3 presents in SGs induced by heat shock, but not in those by glucose starvation.^29^, ^30^ SGs also contain many other components, including RNA helicase, regulators of translation and stability, and factors affecting cell signal transduction.

### 2.2 Factors affecting stress granule assembly

#### 2.2.1 Liquid‐liquid phase separation

**FIGURE 1:**
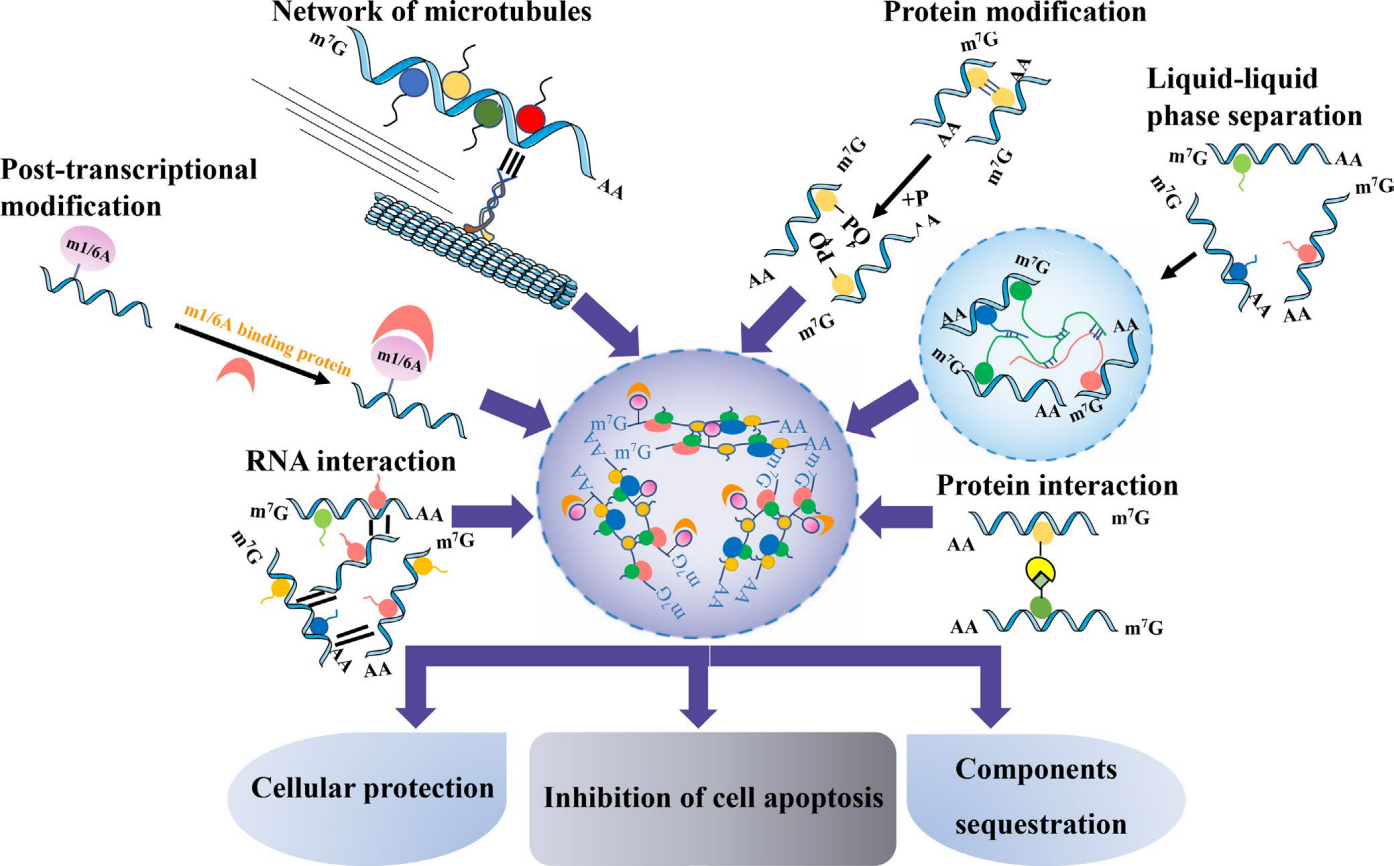
Factors affecting stress granule (SG) assembly and the function of SG. Post‐transcriptional modifications (m^6^A and m^1^A), RNA interaction, the network of microtubules, protein interactions, protein modifications and liquid‐liquid phase separation all impact SG assembly are shown. SGs function as cellular protection, prevent cell apoptosis and sequestrate components. m^6^A, N^6^‐methyladenosine; m^1^A, N^1^‐methyladenosine

Stress granule is a dynamic structure with multiphase properties, which is consistent with the fact that many RNP granules are liquid‐liquid separated.^31^, ^32^ Phase separation describes a phenomenon in which different cell components collide with each other and fuse to form droplets. Some components of the structure are enclosed in the droplets and others are blocked outside the droplets, similar to a mixture of water and oil, which is a common phenomenon in liquids.^33^ Using fluorescence recovery after photobleaching (FRAP) approach, the structural features of P‐bodies (another RNP granule) are characterized. P‐bodies exhibit properties of liquid droplets, which collide and fuse with each other, disperse into smaller droplets after violent vibration, and then can rapidly fuse to form larger droplets.^34^ Recent studies have shown that liquid‐liquid separation (LLPS) is probably the physical and chemical basis for cells to form membraneless organelles such as nucleoli, P bodies, SGs and other distinctive protein/RNA phase transitions.^35^, ^36^ These results seem to be consistent with studies that have uncovered that the process of LLPS is the main driving force to promote the assembly of these structures^34^, ^37^, ^38^ (Figure 1).

The highly disordered domain in RNA‐binding proteins, also known as the low complexity domain, is one of the important molecular characteristics of phase separation. Recent study found that N6‐methyladenosine (m^6^A)‐modified RNA can promote phase separation of YTHDF family proteins in vitro.^39^ YTHDF1, YTHDF2 and YTHDF3 (classical m^6^A‐binding protein) are highly conserved in the structure, containing the binding site of the m^6^A YTH domain and a period of approximately 40 KDa disordered area (low complexity domain). This finding further supports the notion that m^6^A‐ modification regulates protein LLPS in cell.^40^ Unequivocally, these results all confirm the relevance between protein phase separation and low complexity domain.

#### 2.2.2 RNA

##### RNA itself and RNA interactions

Likewise, RNAs have been proven to be required for SG formation. SG assembly increased with stalled translation initiation, whereas decreased when mRNAs are captured by ribosomes. Thus, non‐translating mRNAs are the indispensable components for SG assembly.^41^ Additional evidence has suggested that the formation of SG can be modulated by RNAs, for instance, specifically, injection of naked RNA into the cytosol promotes SG formation.^42^ Similarly, transfection of short RNAs into cells induces the larger foci of SGs.^43^

In addition, interactions between RNA molecules in SG formation have recently drawn intensive attention.^44^, ^45^ The sequence‐specificity and base‐pairing properties of RNAs can induce phase separation of themselves, which may facilitate the assembly of physiological granules.^46^ For example, RNA‐containing G‐quadruplexes (G4) trigger SG nucleation by acting as molecular scaffolds and isolating certain RBP (such as G3BP1)^43^ (Figure 1).

##### Emerging factors—m^6^A, m^1^A modification

Epigenetic mechanisms, such as DNA methylation, RNA methylation and chromatin modification, are involved in adapting to external stimuli under physiological or pathological conditions.^47^, ^48^, ^49^ m^6^A, a common post‐transcriptional modification of RNA, plays a critical role in stress response.^50^ A recent study showed elevated levels of m^6^A after stress exposure and demonstrated that m^6^A plays a pivotal role in selectively sorting mRNAs to SGs.^51^ The m^6^A‐modified RNA signal detected by the specific antibody elevated in a dose‐dependent manner after oxidative stress exposure induced by sodium arsenite.^51^ Alternatively, the integrated stress response (ISR) promotes cellular adaptation to stress conditions through the common target eIF2α. In response to amino acid starvation, the translational reboot of transcription factor 4 (ATF4) was regulated not only by the eIF2‐signalling pathway but also by m^6^A‐modified mRNA. Silencing m^6^A mRNA methylases significantly elevated ATF4 translation efficiency.^52^ Further study has shown that m^6^A modification at 5′ untranslated regions of mRNA can screen ribosomes and subsequently select starting codons.^53^ Together, these studies provide insights into m^6^A function in response to different stress stimuli.

Another epigenetic modification that has not been mentioned that much until recently is N^1^‐methyladenosine (m^1^A). Although N^1^‐adenine (m^1^A) is less popular, it has a significant effect on RNA structure. The methyl group on N^1^‐adenine can interfere with Watson‐Crick base pairing, leading to local duplex melting.^54^ Analysing the motif of mRNAs in SGs revealed a significant enrichment of transcripts targeted by TRMT6/61A (considered as an m1A writer).^55^, ^56^ And further, TRMT6/61A knockout impaired granulation in response to heat shock and arsenite stress, which indicates TRMT61A is involved in RNA granulation under stressful conditions^57^ (Figure 1).

#### 2.2.3 Protein

##### Protein modification

The most common factor regulating SG assembly is protein modification, which modulates both the interaction and function of mRNPs components in SGs. Of the various protein modifications, phosphorylation is well known to function in SG assembly. For instance, phosphorylated eIF2α substantially reduces the assembly of SGs in response to various stress responses including ultraviolet irradiation (UV) and amino acid depletion during translation initiation.^58^ Simultaneously, the aggregation of phosphorylated tristetraprolin (TTP), butyrate response factor (BRF1) and Ras GTPase‐activating protein binding protein (G3BP1) in SGs is reduced when eIF2α is activated.^59^

Acetylation/deacetylation also affects the formation of SGs. The deacetylase activity of SIRT6 (a member of the Sirtuin family of NAD (+)‐dependent enzymes) is essential for G3BP granule formation.^60^ SIRT6 depletion or inhibition by nicotinamide (deacetylase inhibitor) results in decreased size of SGs, whereas overexpression of deacetylase‐disrupted mutant (H133Y, R65A) of SIRT6 cannot rescue the phenotype, which reveals that deacetylase activity is vital for SG formation promoting function.^60^ Moreover, a previous study discovers that histone deacetylases 6 (HDAC6), a cytoplasmic deacetylase, can be recruited to SGs and colocalized with G3BP1 under oxidative stress induced by arsenite and other stress conditions such as UV irradiation, CCCP (mitochondrial stress) and heat shock, which reveals that HDAC6 is a novel critical SG component.^61^ These results are in accord with that HDAC6 modulates acetylation of G3BP1, contributing to the disintegration of SGs.^59^ Based on several reviews, we conclude that HDAC6 is a unique deacetylase consisting of two catalytic domains and a C‐terminal zinc finger domain binding with ubiquitin and ubiquitinated proteins.^62^, ^63^ Further, other investigations have manifested that HDAC6 deacetylates tubulin and microtubule networks.^61^, ^64^, ^65^, ^66^ HDAC6 also binds to ubiquitin to decompose heat‐shock proteins.^67^ Likewise, ubiquitin‐modified proteins are present in SGs. Ubiquitin‐binding domain mutations of HDAC6,^62^ E3 ubiquitin ligase EDD (E3 isolated by differential display), proteasome and other factors related to ubiquitin metabolism can affect the formation of SGs.^68^

Methylation is another important modification of SG‐related proteins. Tudor domain‐containing protein 3 (TDRD3) binds to methyl groups through Tudor motifs that are required for localization of specific SG components.^69^, ^70^ Moreover, protein methylation and Tudor motifs are also associated with the formation of processing bodies and germ cell granules.^21^

Posttranslational modifications of the mRNP components are ideal mechanisms for modulation protein function under stress conditions, because of rapid and reversible protein modifications without new protein synthesis. Elucidating the key physiological purposes of various modifications and the underlying mechanisms of their effects will, therefore, be a valuable goal in the future ^71^ (Figure 1).

##### Protein interaction domain

Based on the analysis of proteomic structural stability of SGs, 50% of the components in SGs are RNA‐binding proteins, which can be absorbed into SGs through protein‐protein interaction.^72^ Accordingly, another factor modulating SG assembly is protein interaction domain in various RNA‐binding proteins. G3BP is a cytoplasmic protein recognized by the SH3 domain that can affect cell cycle, signalling transduction, SG formation and occurrence of some diseases.^73^

Another important finding is that G3BP proteins contain a dimerization domain that contributes to SGs formation under arsenic stress.^74^ In addition, proteins involved in RNA metabolism embody glutamine/asparagine (QN)‐rich domains, which can facilitate SGs assembly through self‐aggregation ability.^75^, ^76^ RNA‐binding proteins T‐cell intracellular antigen‐1 (TIA‐1), T‐cell intracellular antigen‐protein and their homologous proteins with conserved QN‐rich domains have been found in SGs,^77^, ^78^ among which TIA‐1 lacked the QN‐rich domain cannot support the formation of SGs.^75^, ^76^ In contrast, overexpression of the QN‐rich domain of TIA‐1 inhibits the regular assembly of SG and produces basic micro‐aggregates containing endogenous TIA proteins.^79^, ^80^ The role of the QN domain in mRNA metabolism is probably quite extensive since the QN‐rich domain facilitates the formation of p‐bodies, and nearly half of the 107 proteins containing the QN domain have been found to be related to various metabolic processes of RNA, such as transportation, translation and degradation in yeast.^81^

Stress granule assembly is regulated by heat shock proteins whose overexpression inhibits SG formation.^82^ Molecular chaperones are vital in maintaining cell homeostasis under stable protein stress, of which heat shock protein 70 (HSP70) has been shown to be involved in the regulation of SG composition and dynamics.^83^ More recently, HSPBP1 (hsp70‐binding protein 1) is found as a novel component of SGs, and its overexpression can promote SG assembly^84^ (Figure 1).

#### 2.2.4 The network of microtubules in cells

Microtubule networks are also a regulating aspect affecting SG assembly. Microtubule and actin filament networks provide a channel for intracellular mRNA transport, while microtubule motor proteins (kinesin, dynein and myosin) offer carriers on these channels, which are necessary for the appropriate assembly of SGs.^76^, ^85^ Thiamethoxazole, a microtubule‐depolymerization drug, can weaken SG assembly, leading to smaller SG foci.^86^

Stress granule is a highly dynamically changing structure since FRAP analysis indicates a rapid exchange of mRNA and protein in the cytoplasm.^87^ This suggests an active mode of transport in and out of foci mediated by a molecular motor during SG assembly and disassembly. Further analysing the presence of dynein subunits in SGs in a variety of different cell lines, a significant accumulation of dynein intermediate chain and dynein heavy chain in SGs is observed.^87^ Inhibition or knockout of dynein enhances the sensitivity of protease to TIA‐1 polymer, which provides more evidence for the formation mechanism of SGs.^88^

However, the underlying mechanism of microtubules in SG assembly is not fully understood. It can be inferred from the existing results that microtubules can provide a platform for mRNPs and translation initiation factors that are effective in translation, through which they promote the formation of SGs. Once the microtubule structure is destroyed, the formation of SGs is diminished^89^, ^90^ (Figure 1).

## 3 FUNCTIONS OF STRESS GRANULES

Evidence has suggested that SGs can improve cell survival under adverse stress by shutting down intracellular transport, translation (sequester related‐components), and proapoptotic pathways^91^, ^92^, ^93^ (Figure 1).

### 3.1 Cellular protection

Stress granules increase the local concentration of proteins and RNA and disrupt the equilibrium state of molecular interactions, which in turn strengthen the aggregation of SGs and ultimately protect cell survival. Previous observations showed that once cells are infected with viruses, SGs aggregate and activate related antiviral proteins, including retinoic acid‐inducible gene I (rig‐1), PKR, oligoadenylate synthetase (OAS) and ribonuclease L (RNase L), to enhance innate immune response and viral resistance.^94^ To counter the above reactions, viruses employ specific mechanisms, such as degradation of G3BP protein, to prevent the formation of SGs, and subsequently promote their replication and synthesis.^95^

Stress granules withstand reactive oxygen species (ROS) damage in cells to buffer oxidative stress. G3BP1 cooperates with ubiquitin‐specific protease 10 (USP10) to regulate the antioxidant activity of SGs, while USP10 can degrade target proteins after binding to G3BP1. Knockout or overexpression strategies have verified the antioxidant functions of G3BP1 and USP10.^96^ Therefore, SGs play a potential protective role in stress response through anti‐inflammatory and antioxidant effects. More recently, it suggests that MAGE‐B2, a testicular‐specific protein, can increase stress tolerance by inhibiting SG formation, revealing a protective mechanism that resistant to stimulus in a tissue‐specific manner.^12^

### 3.2 Inhibition of cell apoptosis

When cells are exposed to stress, either apoptosis or antiapoptosis can be induced to cope with or repair stress‐induced unfavourable alterations. The cell repair process prevents DNA and proteins from distortion to minimize loss of cell. Cell fate depends on the type and strength of stresses, among which sodium arsenite, low oxygen and heat shock can induce the formation of SGs.^50^, ^97^

It is well known that SG contains factors that are involved in apoptotic regulation; thus, SG could play a role in the apoptotic response. Studies have shown that impaired SG formation is often accompanied by reduced cell viability under stress stimuli.^98^, ^99^ These results are in accord with the notion that SGs cannot be formed when cells encounter endoplasmic reticulum stress (caused by misfolded protein) and oxidative stress (induced by ROS), resulting in promoting cell apoptosis.^100^

The antiapoptotic effect of tumour cells in tumour therapy is related to SG. The underlying mechanism is proposed that SG prevents apoptotic regulatory proteins from interacting with other factors. Chemotherapy drugs promote interaction between the receptors for activated C kinase 1 (RACK1) and mitogen‐activated protein three kinase 4 (MAP3K4), then activate MAP3K4 to mediate cell apoptosis. However, the hypoxic condition can induce SG formation in the tumour cell, which recruits and sequestrates RACK1 in SGs, thus inhibits the activation of MAP3K4 and apoptosis.^10^

### 3.3 Components sequestration

Stress granules sequester intracellular components to block their interactions in the cytoplasm. Previous studies have shown that SGs regulate cell signalling pathways by isolating proteins such as TOR, RACK1 or tumour necrosis factor (TNF) receptor‐associated factor 2 (TRAF2).^101^, ^102^ It has been reported that signalling receptor protein RACK1 is restricted in SGs when cells are exposed to heat stress, thus inhibiting P38 and JNK (c‐Jun N‐terminal kinase) apoptotic signalling pathways.^13^ Moreover, SGs inhibit apoptosis by recruiting the regulatory protein mTOR (mammalian TOR) to block the hyperactivation of the mTOR complex 1 (mTORC1) signalling pathway.^102^ This finding is consistent with the observation that deletion in azoospermia‐like (DAZL)‐containing SGs protect male germ cells from heat stress‐induced apoptosis by sequestering specific signal molecules in SGs, like RACK1, and finally blocks the downstream apoptotic mitogen‐activated protein kinases (MAPK) pathway.^103^ Besides, SGs can segregate proteins related to mRNA physiology and metabolism, causing temporary translation inhibition and thus preventing the accumulation of misfolded proteins.^104^

## 4 STRESS GRANULES INVOLVING IN BIOLOGICAL DISORDERS

### 4.1 Male fertility

It is well established that thermal stress indeed affects the fertility of male animals. In most mammals, the testicles are located in the scrotum outside the body cavity, where spermatogenesis usually occurs. Therefore, exogenous and endogenous forms of insults (eg high temperature) affect mammalian spermatogenesis and ultimately lead to subfertility and even infertility.^105^ Offspring from male mice with a heat‐treated scrotum mated with normal female mice, and exhibited lower weight than those from males without heat treatment.^106^ Studies have shown that oxidative stress is a leading outcome of heat damage in spermatogenic cells,^1^, ^107^ while sperms and oocytes are the most sensitive to heat,^108^, ^109^, ^110^ and the somatic supporting cells such as Sertoli cell in the testis are also affected.^111^, ^112^

Unlike somatic cells, the germline has its unique functions and characteristics, the most important of which transmits genetic information accurately from generation to generation.^113^ In order to produce viable offspring, germline must be able to cope with all kinds of environmental pressures. The testicles of most mammals, where spermatogenesis occurs, situate the scrotum outside the body cavity and affect by ambient temperature. The scrotum temperature is ordinarily 2‐7 degrees, lower than the body's core temperature. Several reports have shown that exposure to heat stress eventually leads to DNA breakage and apoptosis in germ cells.^114^, ^115^, ^116^, ^117^, ^118^ The lower temperature is essential for normal spermatogenesis, as remarkable germ cell loss has been found in cryptorchidism and testes treated by heat.^119^, ^120^

Very little is currently known about the molecular mechanism that protects spermatogenesis from adverse temperature fluctuation; however, SG provides new insights into the male reproductive field.^121^, ^122^, ^123^ In addition, RNA‐binding proteins are required for natural fertility in germ cells.^124^ Previous research has shown that the reduction of RNA‐binding protein expression (DAZL, DAZ, BOULE) leads to infertility in mammals.^125^, ^126^ Upon identifying two gene families on the Y chromosome of humans, RBMY and DAZ, it is found that the deletion of either was associated with the failure of germ cells during spermatogenesis. Another important finding is that DAZL can colocalize with TIA1, an SG marker in HeLa cells during oxidative stress, which indicates that DAZL will be recruited in SGs.^127^ Accordingly, DAZL is a necessary element of SGs in mouse germ cells upon heat stress, which confirms previous studies.^103^ A recent study demonstrates that MSI‐1, an mRNA‐binding protein, functions as modulating the fate of Sertoli cells after heat‐induced damage and plays an important role in supporting spermatogenesis ^117^ (Table 1).

**TABLE 1:** Overview of components involved in stress granules in germ cells

Components	Function	References
DAZL	Prevent male germ cells from undergoing apoptosis upon heat stress	^99^
TIAR‐1	Promote fertility and embryonic development	106, 114
EIF2α	As a protective mechanism against heat stress in mouse male germ cells	^119^
BOULE	As conserved germ cell‐specific translational regulators	^118^
NANOS2	Stabilized NANOS2 may be responsible for the reduction of the spermatogonial progenitor cell (SPC) pool	^117^
MUSASHI‐1	Critical for constructing a functional BTB structure and maintaining spermatogenesis; regulating Sertoli cell fate following heat‐induced injury	^113^
DZIP1	Important for the formation of stress granules during the stress response	^120^
MAGE‐B2	Increase stress tolerance by inhibiting SG formation	^12^

**FIGURE 2:**
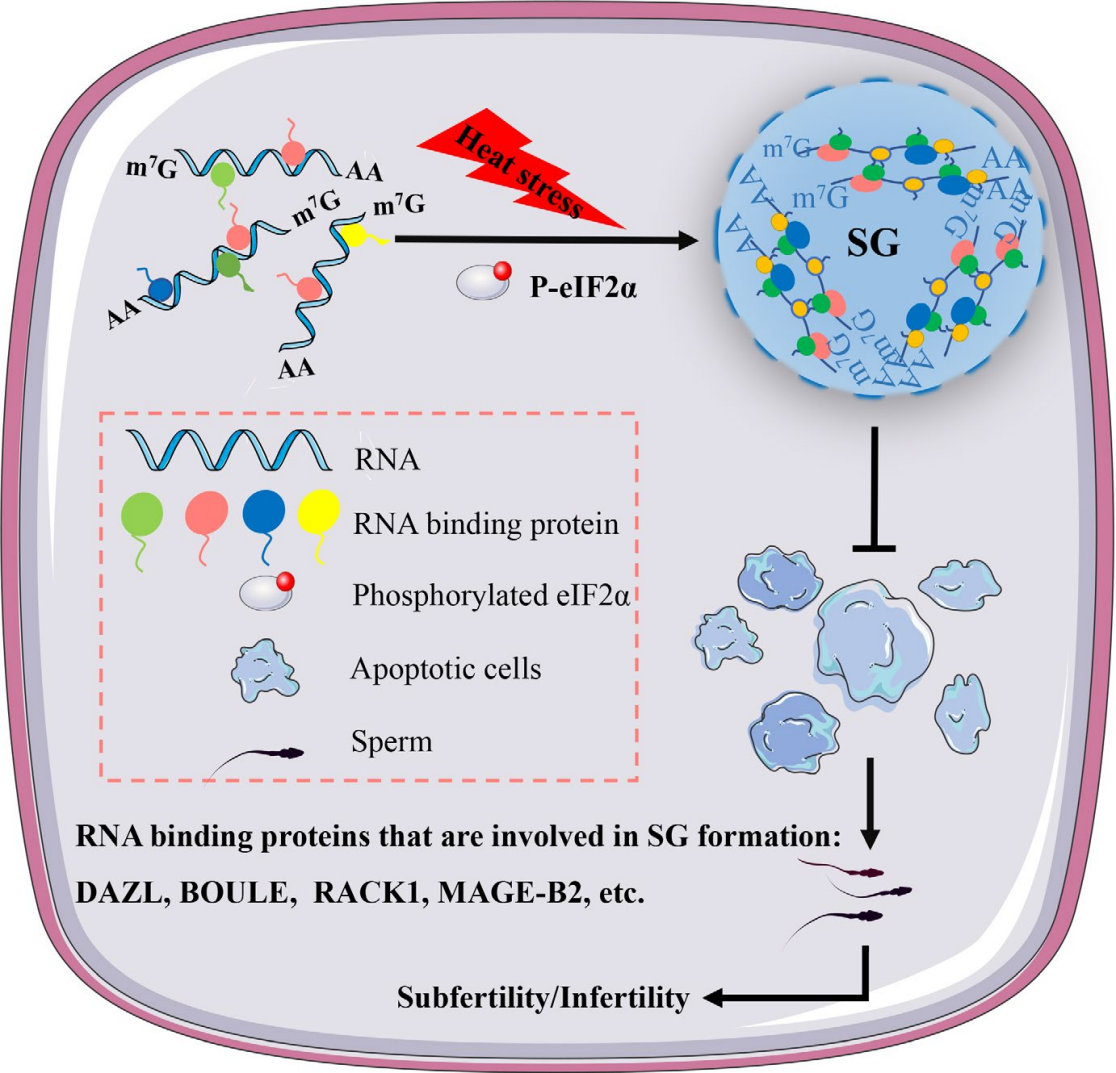
Schematic illustration for heat stress in male germ cells. Heat stress induces stress granule (SG) formation by phosphorylating eIF2α, recruiting RNA binding protein (DAZL, BOULE, RACK) and ultimately protecting normal spermatogenesis from germ cell apoptosis. In addition, MAGE‐B2, a testis‐specific protein, can enhance stress tolerance by modulating SG formation. BOULE, a founding member of the DAZ gene family; DAZL, deleted in azoospermia‐like; eIF2α, eukaryotic initiation factor‐2alpha; MAGE‐B2, testis‐specific protein; MAPK, mitogen‐activated protein kinases; RACK, receptor for activated protein kinase C

Apart from heat stimuli, high concentrations of glucose have been shown to induce the assembly of RNP particles in the germline of *C elegans*, and further studies suggest that this process is mediated by the osmotic pressure response. They also find that destruction of RNP particle assembly is associated with reduced oocyte mass in meiotic‐block.^128^ This indicates that the assembly of RNP particles in germ cells prevents mRNA degradation or early translation for maintaining oocyte quality.^129^, ^130^ Reviewing how the male genital line reacts to stressors, particularly the assembly and function of SGs, could ultimately improve our understanding of human fertility and provide insights into the role of related RNP complexes in other types of cells (Figure 2).

### 4.2 Stress granules in other biological processes

#### 4.2.1 Inflammatory response

**FIGURE 3:**
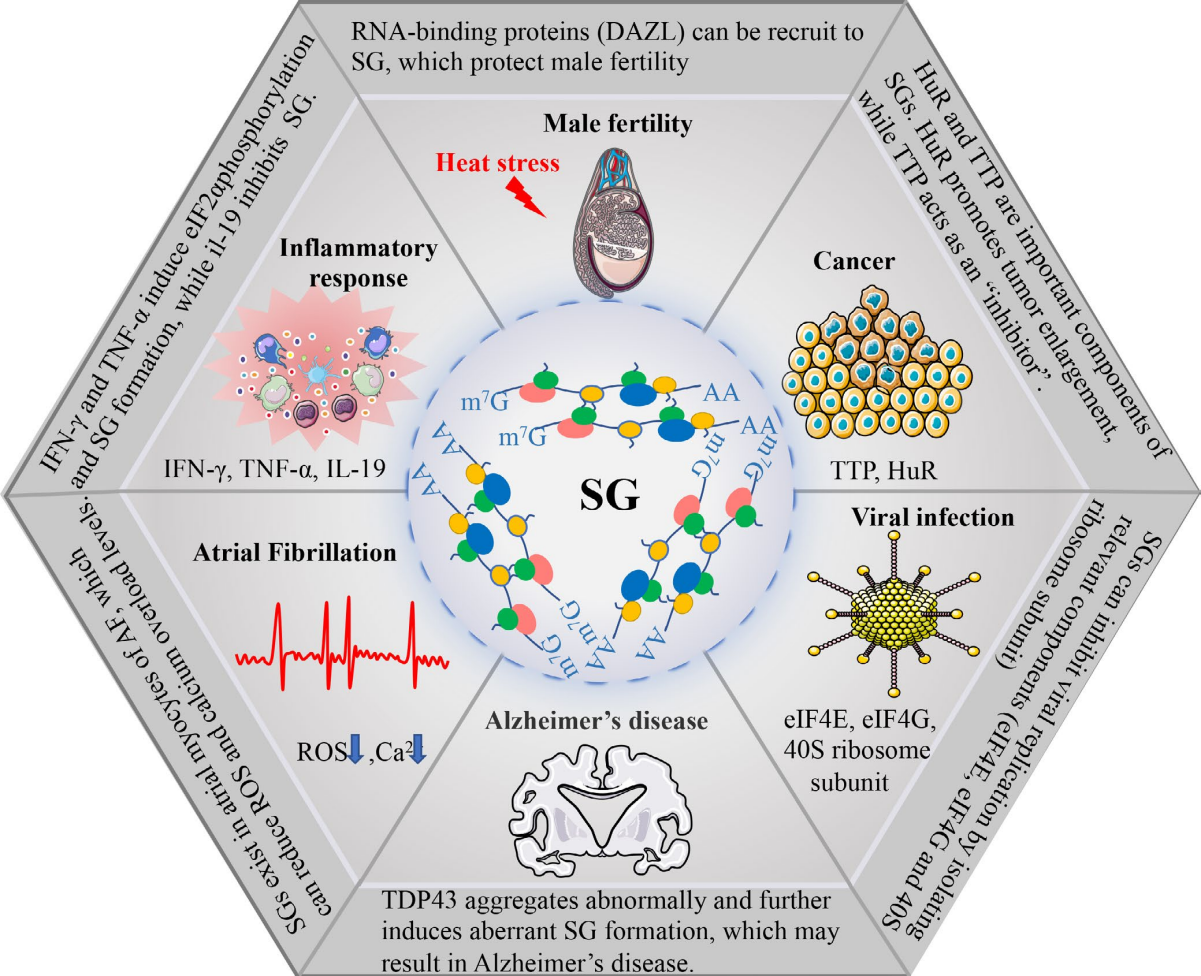
Areas in which stress granules (SGs) are involved. SGs function in male fertility and play roles in other biological and pathological processes, such as inflammatory response, Alzheimer's disease, viral infection, cancer and atrial fibrillation. Spermatogenesis is affected by heat stress in the testicles outside the body cavity. RNA‐binding proteins (DAZL) can be recruit to SGs, which protect male fertility. Pro‐inflammatory cytokines (IFN‐γ and TNF‐α) induce eIF2α phosphorylation and SGs formation, while anti‐inflammatory cytokine (il‐19) inhibit the formation of SG. Misfolded RNA binding proteins (such as TDP43) aggregate abnormally and further induce aberrant SG formation, which results in Alzheimer's disease. SGs can inhibit viral replication by isolating relevant components (eIF4E, eIF4G and 40S ribosome subunit). HuR and TTP are important components of SGs. Overexpression of HuR in cancer cells leads to tumour enlargement, while TTP plays an anti‐tumour role. SGs exist in atrial myocytes of AF, which can reduce ROS and calcium overload levels. AF, Atrial fibrillation; eIF4E, eIF4G, eukaryotic translation initiation factor 4E/G; HuR, Hu antigen R; IFN‐γ, interferon; il‐19, interleukin‐19; ROS, reactive oxygen species; TDP43,TAR DNA‐binding protein 43; TNF‐α, tumour necrosis factor alpha; TTP, tristetraprolin

Inflammatory factors are directly or indirectly associated with SG formation. In mucosal inflammation, the pro‐inflammatory cytokines interferon (IFN)‐γ and TNF‐α induce phosphorylation of eIF2 to form SGs, encapsulating HSP70 mRNA into SGs and thus reducing HSP70 translation.^16^ SGs caused by heat shock recruit TRAF2 and inhibit TNF‐α‐mediated NF‐κB activation by interacting with eIF4G.^131^ Since environmental stimuli can trigger an inflammatory response, SG‐related proteins may be associated with the inflammatory response. Emerging evidence has shown that eIF2α phosphorylation increased and SGs formed upon exposure to stimuli, which can be reversed by treating the anti‐inflammatory cytokine interleukin‐19^132^ (Figure 3).

#### 4.2.2 Degenerative disease

Misfolded proteins and mutations in RNA‐binding proteins, such as TAR DNA‐binding protein 43 (TDP43), are responsible for many types of neurodegenerative diseases, like Alzheimer's disease^133^ and amyotrophic lateral sclerosis.^134^, ^135^ Mutations in RNA‐binding proteins boost self‐assembly, which leads to the formation and persistence of SGs.^23^, ^136^ Under normal conditions, autophagosomes play an important role in clearing SGs, but an aggregation of mutated proteins (optic nerve protein, ubiquitin‐2, etc) in SGs seriously impairs autophagy function, leading to degenerative diseases of muscles and nerves^137^ (Figure 3).

#### 4.2.3 Viral infection

Viral infections trigger a stress response and lead to the formation of SGs. Pattern recognition receptors, such as RIG‐I‐like receptors (RLRs), which detect non‐native RNA in virus‐infected cells and produce antiviral agents, play a crucial role in clearing invading viruses. It has been shown that when infected with a variety of viruses, RLRs, mRNAs, 40S ribosome subunits and RNA‐binding proteins are colocalized in the virus‐induced SGs.^138^ IFN is significantly reduced via artificially suppressing the formation of SGs induced by viruses, which indicates that SGs play a vital role in innate antiviral immune.^138^ Translation initiation factors, such as eIF4E, eIF4G and the 40S ribosome subunit in SGs, are essential for virus translation and replication. SGs can inhibit viral replication by isolating these components ^139^, ^140^ (Figure 3).

#### 4.2.4 Cancer

RNA‐binding proteins in SGs regulate cancer‐associated target mRNAs.^18^, ^19^, ^141^ eIF4E expression and activity elevate approximately 30% in different malignancies, and its overexpression is associated with poor prognosis, especially in malignant hematopathy.^18^, ^19^ Meanwhile, eIF4E is an essential component of SGs. Whether mRNA that can bind to eIF4E is preferentially recruited to SGs remains to be further investigated. Hu antigen R (HuR) and TTP proteins, which are important components of SGs, have opposite effects on target mRNAs. HuR stabilizes the transcription and regulates the translation process, while TTP promotes the degradation of target mRNAs. A study suggests that TTP plays an anti‐tumour role, and its expression is negatively correlated with the progression of breast and prostate cancer.^141^ In the xenograft model of mice, overexpression of HuR in tumour cells leads to tumour enlargement, while its depletion leads to reduced tumour volume.^142^ Therefore, SGs likely play functions in tumour progression (Figure 3).

#### 4.2.5 Others

Retinitis pigmentosa (RP) is a degenerative disease of the retina. Ceramide kinase‐like (CERKL) can cause RP and cone malnutrition, while it is also an important component of SGs. The absence of SGs is associated with pathological mutations in CERKL. CERKL is also associated with microtubules and has been found in neurites of neuromutant cell lines. Therefore, the correlation between RP and SGs is the key to study its pathological mechanism and treatment.^143^

Atrial fibrillation (AF) is the most common arrhythmia in clinical practice, in which chronic inflammatory response and oxidative stress play an important role. It has been confirmed that SGs exist in atrial myocytes of AF and can reduce ROS and calcium overload levels.^144^ However, whether SGs can fight against apoptosis and fibrosis, thus reducing the AF incidence, remains unknown. Consequently, it is of great significance to further reveal the aetiology and potential therapeutic targets of AF (Figure 3).

## 5 CONCLUDING REMARKS

Assembly defect of SGs is the cause of many diseases and abnormal physiological processes. Existing findings have remarkable implications for understanding how cells react to the environmental stimulus through SGs formation. As a typical membrane‐free organelle, SGs have highly scientific significance. The synthesis and functional study of SGs is a promising novel field in cell biology. However, SGs have dynamic formation and depolymerization characteristics, which makes it challenging to study their details. How mRNAs locate in the different subcellular chambers and how post‐transcriptional regulation affects mRNA translation and degradation remain further research. In particular, there are relatively few studies on SGs in the reproductive field, and therefore, future investigations need to be enhanced from these aspects.

## CONFLICT OF INTEREST

The authors declare no conflict of interest.

## AUTHOR CONTRIBUTIONS

LW, WY and BL reviewed the literature and drafted the manuscript. FW and SY revised the manuscript. All authors have approved the current version of the manuscript.

## Data Availability

Data sharing is not applicable to this article as no new data were created or analysed in this study.
